# The Psychological Impact of COVID-19 on Healthcare Workers in Saudi Arabia: A Year Later Into the Pandemic

**DOI:** 10.3389/fpsyt.2021.797545

**Published:** 2021-12-17

**Authors:** Atiah H. Almalki, Mohammad S. Alzahrani, Fahad S. Alshehri, Adnan Alharbi, Samirah F. Alkhudaydi, Rawan S. Alshahrani, Aseel H. Alzaidi, Majed A. Algarni, Hashem O. Alsaab, Yasser Alatawi, Yusuf S. Althobaiti, Ahmed K. Bamaga, Abdullah A. Alhifany

**Affiliations:** ^1^Department of Pharmaceutical Chemistry, College of Pharmacy, Taif University, Taif, Saudi Arabia; ^2^Addiction and Neuroscience Research Unit, Health Science Campus, Taif University, Taif, Saudi Arabia; ^3^Department of Clinical Pharmacy, College of Pharmacy, Taif University, Taif, Saudi Arabia; ^4^Department of Pharmacology and Toxicology, College of Pharmacy, Umm Al-Qura University, Makkah, Saudi Arabia; ^5^Department of Clinical Pharmacy, College of Pharmacy, Umm Al-Qura University, Makkah, Saudi Arabia; ^6^College of Pharmacy, Taif University, Taif, Saudi Arabia; ^7^Department of Pharmaceutics and Pharmaceutical Technology, Taif University, Taif, Saudi Arabia; ^8^Department of Pharmacy Practice, Faculty of Pharmacy, University of Tabuk, Tabuk, Saudi Arabia; ^9^Department of Pharmacology and Toxicology, College of Pharmacy, Taif University, Taif, Saudi Arabia; ^10^Neurology Division, Pediatric Department, Faculty of Medicine, King Abdulaziz University Hospital, King Abdulaziz University, Jeddah, Saudi Arabia

**Keywords:** mental health, Saudi Arabia (KSA), COVID-19, health care workers (HCW), depression, anxiety, stress

## Abstract

**Objective:** The COVID-19 pandemic poses unprecedented challenges to healthcare workers worldwide. This study sought to estimate the prevalence of depression, anxiety, and stress among healthcare workers in Saudi Arabia, and to identify the factors associated with these psychological disorders.

**Methods:** A cross-sectional questionnaire-based study was conducted from January 21 to March 2, 2021. Physicians, pharmacists, nurses, and other healthcare workers from different parts of Saudi Arabia were recruited through snowball sampling. Psychological outcomes were measured using the Depression, Anxiety, and Stress Scale (DASS-21). Pearson's chi-square test was used to explore the bivariate association between diverse characteristics and each outcome. Multiple logistic regression analyses were performed to identify factors associated with depression, anxiety, and stress.

**Results:** A total of 501 healthcare workers completed the survey, of whom 60% were female and nearly half were pharmacists. The majority (76.25%) of respondents reported that a family member, friend, or colleague had contracted COVID-19, and more than one-third (36%) knew someone who died due to COVID-19. Overall, the estimated prevalence rates of depression, anxiety, and stress were 54.69, 60.88, and 41.92%, respectively. The multivariate analysis revealed that healthcare workers with chronic diseases, nurses, and healthcare workers from the southern region were more likely to suffer from depression and stress. Further, individuals with positive COVID-19 test results showed a greater proportion of depressive symptoms compared to others. In addition, knowing someone who died due to COVID-19 and having a chronic illness were predisposing factors for anxiety.

**Conclusion:** After more than a year, the prevalence of depression, anxiety, and stress remains substantial among healthcare workers in Saudi Arabia. The findings can help guide efforts to mitigate the psychological impact of the pandemic.

## Introduction

The novel coronavirus (SARS-CoV-2) was first reported by the Chinese government in December 2019 in the city of Wuhan (1). and declared a pandemic by the World Health Organization (WHO) on March 7, 2020 (2). SARS-CoV-2 has caused a similar pathogenesis as previous coronaviruses, such as Middle East Respiratory Syndrome (MERS) in 2012 (3). Pandemics and natural disasters often have a psychological impact on infected people and those in direct contact, such as healthcare workers (HCWs). About 30% of the general population in China has complained of moderate to severe anxiety (4). A study conducted among students found that anxiety and stress were highly associated with academic delays and low quality of life during the corona pandemic (5). Moreover, many medical doctors and nurses in Wuhan reported mental disturbances and anxiety disorders, and indicated that mental health support provided relief and alleviated symptoms (6, 7). In China, the incidence of psychological stress and anxiety in medical staff was higher compared to college students, possibly explained by the student's limited contact with confirmed positive COVID-19 cases in comparison to practicing healthcare providers (8).

Unlike prior outbreaks, the COVID-19 pandemic could have a potentially long-term psychological impact on both the general public and HCWs. One year following the start of the pandemic, few studies have been conducted to assess the psychological impact among HCWs using the Depression, Anxiety, and Stress Scale (DASS-21) questionnaire (9–15). Also, few longitudinal studies have been conducted to evaluate the long-term impact of the pandemic on health care providers (16–22). A recent Chinese study reported that some residents were still suffering from depression and anxiety during the low transmission period one year after the start of the pandemic (23). As Saudi Arabia also enters the low transmission phase, a timely psychological assessment is needed to identify vulnerable populations.

To date, few studies have evaluated the long-term impact of COVID-19 on the Saudi population, and none have tackled the long-term impact on HCWs in Saudi Arabia (3, 24, 25). The HCWs were on the front line of the crisis in Saudi Arabia, whether in hospitals or community pharmacies. Few studies have assessed the psychological impact of the COVID-19 pandemic among HCWs in Saudi Arabia in the past year (11, 26, 27), and no study has examined pharmacists working on the front line of this crisis, whether in hospitals or community pharmacies, or while delivering patient medications. The present study, therefore, aimed to investigate the long-term impact of COVID-19 among pharmacists and other HCWs one year following the start of the pandemic.

## Methods

### Study Design and Setting

A cross-sectional questionnaire-based study was conducted from January 21 through March 2, 2021. HCWs residing in Saudi Arabia and working at the Saudi MOH, local community pharmacy chains, and other government or private hospitals were eligible to participate in the study. Exclusion criteria included those under 18 years of age, non-Arabic speakers, and non-HCWs. The study participants were recruited using a snowball sampling technique, where a link to the online survey was promoted and shared via WhatsApp, Twitter, and internal emails. The web-based survey was designed to ensure that every participant could take part only once. All participants were informed of the study purpose and were assured of the confidentiality of their responses. The study was approved by the Scientific Research Ethics Committee at Taif University (42-0068) and the Institutional Review Board of MOH (472).

A priori sample size was calculated according to the formula suggested by Lwanga and Lemeshow (28). We assumed that the prevalence of psychological distress during the COVID-19 pandemic in Saudi Arabia was 23.6%, as reported in a recent study (29). The required sample size was calculated using OpenEpi (Version 3.01, Atlanta, USA). The minimum sample size required for 80% power was 416, with a 95% confidence level, 5% confidence limit, and 1.5 design effect.

### Data Collection

Data were collected via a standardized, self-administered questionnaire. We adopted the Depression, Anxiety, and Stress Scale−21 (DASS– 21), a reliable and valid self-administered instrument to screen for these psychological disorders (30). This survey tool has 21 items, each of which is scored on a scale from 0 (“does not apply to me at all”) to 3 (“applies to me most of the time”). Scores for each subscale are determined by summing the scores of relevant items and then multiplying by a factor of 2. Each subscale was categorized into normal, mild/moderate, and severe/extremely severe based on the recommended cut-off values (30). The survey tool was translated to Arabic according to the forward and backward translation technique, which is known as a cross-cultural adaptation of research instruments (31). The process included two steps: (1) the forward translation from English to Arabic by two translators fluent in Arabic and English; and (2) the backward translation from Arabic to English by two different translators also fluent in both languages. Afterward, two faculty members with knowledge of the subject assessed the face validity of the Arabic version of the questionnaire.

We gathered demographic and occupational characteristics from the questionnaire, including data on age, gender, nationality (Saudi or non-Saudi), marital status (married or unmarried), and geographic region (central, eastern, western, northern, or southern region). Job occupation was categorized into physician, pharmacist, nurse, and others, which included various occupations such as dentist, laboratory worker, radiology technician, and medical intern. In addition, we collected data on whether the participants had a chronic illness, whether they had tested positive for COVID-19, and whether someone they knew had tested positive for COVID-19 or had died due to COVID-19.

### Statistical Analysis

Univariate, bivariate, and multivariate statistical analyses were conducted. Frequency and percentages were used to describe characteristics and estimate prevalence rates of depression, anxiety, and stress among participants. A bivariate analysis was conducted using Pearson's chi-square test to explore the association between sociodemographic traits and each DASS subscale. Variables that were significantly associated with the outcomes were further analyzed by entering the adjusted multivariate model. Age, gender, and job category were predetermined to enter into the adjusted model, regardless of their bivariate association with each outcome. A multivariate logistic regression analysis determined the factors associated with each outcome (i.e., depression, anxiety, and stress). Adjusted odds ratios with 95% confidence intervals (CIs) and *p*-values were calculated to determine the strength and significance of the association. All statistical analyses were performed using SAS software (version 9.2, SAS Institute Inc., Cary, NC, USA).

## Results

A total of 501 HCWs completed the survey, nearly half of whom were pharmacists (*n* = 244). The response rate was 97.8%. The majority of participants were Saudis, one-third were from the Western region, and about 60% were female (Table 1). While only 18.76% of the participants had tested positive for COVID-19, the majority (76.25%) reported that a family member, friend, or colleague had tested positive for COVID-19. In addition, about 44% of physicians reported that a family member, friend, or colleague had died due to COVID-19, compared to 36.07 and 32.08% of pharmacists and nurses, respectively.

**Table 1:** Sociodemographic characteristics of study participants.

**Characteristic**	**Job category**				**Total** **(*N* = 501)**
	**Physicians** **(*N* = 63)**	**Pharmacists** **(*N* = 244)**	**Nurses** **(*N* = 54)**	**Others** **(*N* = 140)**	
Female, %	68.25	44.67	79.63	78.57	60.88
Age, % 18–24 25–34 35 or older	38.10 38.10 23.81	30.33 56.97 12.70	55.56 33.33 11.11	59.29 28.57 12.14	42.12 44.11 13.77
Nationality, % Saudi Non-Saudi	61.90 38.10	92.21 7.79	83.33 16.67	85 15	85.43 14.57
Marital status, % Married Single/widowed/divorced	30.16 69.84	35.80 64.20	24.07 75.93	18.57 81.43	29.00 71.00
Region, % Central Region Eastern Region Northern Region Southern Region Western Region	41.27 17.46 9.52 11.11 20.63	25.82 11.07 7.79 13.52 41.80	35.85 32.08 7.55 9.43 15.09	30.94 15.11 10.79 12.23 30.94	30.26 15.23 8.82 12.42 33.27
Healthcare setting, % Inpatient Hospital Setting Outpatient Hospital Setting Primary Health Care Centre Community Pharmacy Other	41.27 12.70 25.40 - 20.63	26.64 15.16 6.15 22.95 29.10	51.85 5.56 16.67 1.85 24.07	31.16 10.87 19.57 - 38.41	32.46 12.63 13.43 11.42 30.06
Have chronic illness, %	28.57	13.52	22.22	19.29	17.96
Tested positive for COVID-19, %	31.75	15.16	24.07	17.14	18.76
Family member, friend, or colleague tested positive for COVID-19, %	74.60	79.51	66.67	75.00	76.25
Family member, friend, or colleague died due to COVID-19, %	44.44	36.07	32.08	33.57	36.00

**Figure 1:**
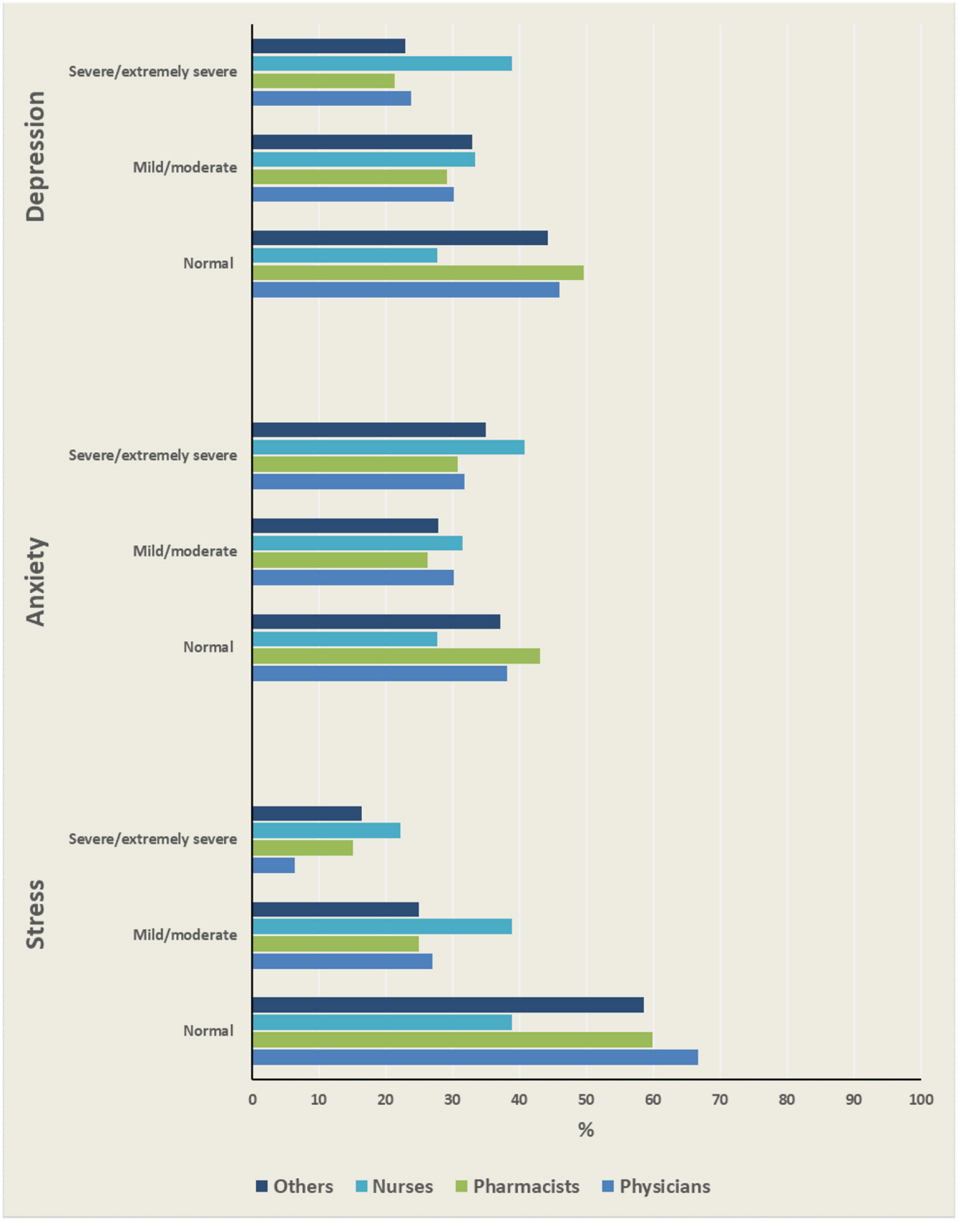
Severity levels of psychological disorders among HCWs.

Based on the DASS subscale scores, the estimated prevalence rates of depression, anxiety, and stress among participants were 54.69, 60.88, and 41.92%, respectively. In terms of severity, one-third (33.13%) of the participants suffered from severe or extremely severe anxiety, 23.95% suffered from severe or extremely severe depression, and 15.17% suffered from severe or extremely severe stress. Stratified by occupation, nurses had the highest severity rates across all DASS subscales (Figure 1). The prevalence rate of stress, ranging from mild to extremely severe, was higher among pharmacists (40.16%) compared to physicians (33.33%), but less than that of nurses (61.11%) (Table 2). The prevalence of depression was significantly higher among participants with chronic diseases and those who had tested positive (*p* < 0.001 and *p* = 0.004, respectively). Chronic illness was also significantly associated with anxiety and stress. The younger age group (18–24 years) had a significantly higher percentage of anxiety and depression than the older age groups. HCWs who were not married had significantly higher rates of depression, anxiety, and stress. Compared to other regions, the southern region of Saudi Arabia had the highest prevalence rates of depression, anxiety, and stress among HCWs.

**Table 2:** Bivariable analysis of depression, anxiety, and stress.

**Characteristic**	**Depression^**†**^, %**	*P-*value^*^	**Anxiety^**‡**^, %**	*P-*value^*^	**Stress^**§**^, %**	*P-*value^*^
Gender Male Female	49.49 58.03	0.06	56.63 63.61	0.12	37.76 44.59	0.13
Age 18–24 25–34 35 or older	65.40 47.51 44.93	<0.001	70.62 54.30 52.17	0.001	47.39 39.82 31.88	0.05
Married Yes No	43.45 59.15	0.001	50.34 65.07	0.002	33.79 45.07	0.02
Nationality Saudi Non-Saudi	56.07 46.58	0.13	61.92 54.79	0.25	42.76 36.99	0.36
Job category Physician Pharmacist Nurse Others	53.97 50.41 72.22 55.71	0.04	61.90 56.97 72.22 62.86	0.19	33.33 40.16 61.11 41.43	0.02
Region Central Region Eastern Region Northern Region Southern Region Western Region	60.26 47.37 43.18 70.97 50.00	0.007	64.24 56.58 59.09 72.58 56.02	0.16	47.02 34.21 40.91 58.06 34.94	0.009
Healthcare setting Inpatient Hospital Setting Outpatient Hospital Setting PHC Community Pharmacy Other	51.85 57.14 53.73 52.63 57.33	0.88	61.73 57.14 61.19 59.65 61.33	0.98	40.74 38.10 41.79 49.12 42.00	0.79
Chronic illness Yes No	75.56 50.12	<0.001	81.11 56.45	<0.001	63.33 37.23	<0.001
Tested positive for COVID-19 Yes No	68.09 51.60	0.004	68.09 59.21	0.11	45.74 41.03	0.40
Family member, friend, or colleague tested positive for COVID-19 Yes No	56.11 54.06	0.66	59.69 64.71	0.33	38.74 52.10	0.009
Family member, friend, or colleague died due to COVID-19 Yes No	52.62 61.34	0.09	67.78 57.19	0.02	42.78 41.56	0.79

† *Depression was defined as DASS-21 depression subscale score ≥ 10*.

‡ *Anxiety was defined as DASS-21 anxiety subscale score ≥ 8*.

§ *Stress was defined as DASS-21 stress subscale score ≥ 15*.

* *P-values produced using Pearson's Chi square test*.

The multivariate analysis of depression showed that nurses had more than two times the odds of suffering from depression compared to physicians (OR = 2.37, 95% CI: 1.03–5.47) (Table 3). In addition, HCWs in the southern region were twice as likely to suffer from depression than their counterparts working in the western region (OR = 2.32, 95% CI: 1.21–4.47). Having a chronic illness and testing positive for COVID-19 were significant predictors of depression. The multivariate analysis of anxiety revealed that the youngest age group (18–24 years old) had greater odds of having anxiety than the oldest age group (35 years or older) (Table 3). The adjusted model indicated that having a chronic illness and knowing someone who died due to COVID-19 were significant predictors of anxiety. Finally, the multivariate analysis of stress suggested that nurses, HCWs in the southern region, and participants with chronic illnesses had significantly greater odds of suffering from stress (Table 3).

**Table 3:** Multivariable logistic regression analysis of depression, anxiety, and stress.

**Characteristic**	**Depression**		**Anxiety**		**Stress**	
	**Adjusted OR** **(95% CI)**	***P-*value**	**Adjusted OR** **(95% CI)**	***P-*value**	**Adjusted OR** **(95% CI)**	***P-*value**
Gender						
Male Female	Ref 1.15 (0.74–1.78)	0.53	Ref 0.95 (0.62–1.46)	0.81	Ref 1.18 (0.77–1.83)	0.45
Age						
35 or older 25–34 18–24	Ref 1.07 (0.56–2.02) 1.71 (0.83–3.52)	0.34 0.06	Ref 1.16 (0.62–2.16) 1.88 (0.92–3.87)	0.42 0.03	Ref 1.33 (0.67–2.61) 1.38 (0.65–2.93)	0.59 0.49
Married						
No Yes	Ref 0.79 (0.47–1.35)	0.39	Ref 0.72 (0.43–1.21)	0.21	Ref 0.83 (0.48–1.43)	0.50
Job category						
Physician Pharmacist Nurse Others	Ref 1.14 (0.61–2.12) 2.37 (1.03–5.47) 1.11 (0.58–2.13)	0.38 0.02 0.34	Ref 0.95 (0.51–1.76) 1.71 (0.74–3.94) 0.99 (0.51–1.89)	0.30 0.11 0.46	Ref 1.78 (0.94–3.38) 3.49 (1.55–7.90) 1.58 (0.81–3.09)	0.98 0.005 0.52
Region						
Western Region Central Region Eastern Region Northern Region Southern Region	Ref 1.24 (0.77–1.99) 0.72 (0.39–1.30) 0.81 (0.39–1.65) 2.32 (1.21–4.47)	0.52 0.06 0.25 0.003	—	—	Ref 1.43 (0.89–2.32) 0.85 (0.46–1.57) 1.27 (0.62–2.60) 2.29 (1.23–4.29)	0.53 0.07 0.95 0.01
Chronic illness						
No Yes	Ref 2.62 (1.52–4.52)	<0.001	Ref 2.93 (1.64–5.24)	<0.001	Ref 2.90 (1.76–4.79)	<0.001
Tested positive						
No Yes	Ref 1.79 (1.07–2.99)	0.03	—	—	—	—
Family member, friend, or colleague died due to COVID-19						
No Yes	—	—	Ref 1.60 (1.07–2.39)	0.02	Ref 0.69 (0.44–1.07)	0.09

## Discussion

The present study examines the mental health toll of the COVID-19 pandemic on HCWs in Saudi Arabia. According to Saudi Commission For Health Specialties (SCFHS) report in 2020, ~500,000 HCWs were registered in the country (32). To our knowledge, this study is among the first to determine the prevalence and associated factors of depression, anxiety, and stress among HCWs in all regions of Saudi Arabia. Our findings indicate a considerably high prevalence rate of psychological disorders among physicians, pharmacists, nurses, and other HCWs during the pandemic. The overall prevalence of depression, anxiety, and stress was 54.69, 60.88, and 41.92%, respectively. The prevalence rates of psychological disorders found in this study were greater than those of the general public in Saudi Arabia revealed in a previous study (29). Several factors can increase the risk of mental health conditions among HCWs, including fear of infection, high workload, and recurrent isolation from family members (33). Findings from this study underscore the importance of mental health interventions for HCWs. Mental health policy makers at health institutions should implement training in coping strategies and stress management skills for their HCWs. In addition, mental health programs and initiatives in Saudi Arabia should promote and expand their mental health counseling services.

The unprecedented pandemic has likely had an inevitable and enduring impact on the psychological well-being of HCWs. In terms of severity, we found that one-third of the HCWs had severe or extremely severe anxiety. This rate was higher compared to previous studies conducted during the early stages of the pandemic among HCWs in Saudi Arabia and other countries (11, 34–36). Our study was conducted in early 2021, a period when the number of cases in Saudi Arabia had reached more than 360,000, and deaths due to COVID-19 had surpassed 6,000 (37). In our sample, more than one-third (36%) of HCWs knew someone who had died due to COVID-19. Our findings suggest that the psychological impact of the pandemic has persisted among HCWs, even though many restrictions in Saudi Arabia have been eased. This is in line with findings observed in a systematic review of previous infectious disease epidemics, such as MERS, suggesting long-lasting effects (38). Longitudinal studies are better suited to investigate the long-term psychological impact of the COVID-19 pandemic. One year later, few studies have been conducted to assess the psychological impact among HCWs using the DASS-21 questionnaire (9–15), and few cohort studies have evaluated the impact of the pandemic on health care providers (16–22). For instance, one study conducted over 3 months showed that improving workplace support might protect HCWs from adverse psychological consequences (39). A study conducted in Singapore among Medical residents found that HCWs were at high risk of psychological sequelae (40). Another study measured the long-term psychological impact of COVID-19 on frontline doctors in the UK over three periods of time (41).

As expected, we found that nurses were most affected by the COVID-19 crisis. Our findings indicate that nurses have a higher prevalence of moderate to severe psychological disorders than other healthcare professionals. Additionally, multivariate analyses revealed that nurses were more likely to suffer from depression and anxiety. This is not surprising because the literature has shown that, even prior to the COVID-19 pandemic, nurses and HCWs in direct contact with patients have a higher risk of depression, anxiety, and sleep disorders (42–44). During the COVID-19 crisis, several studies reported that nurses were more vulnerable to psychological disorders than other HCWs (11, 34, 45). Compared to other professionals, nurses may be at greater risk of contracting COVID-19 because of their regular and close contact with patients.

In this study, we explored the association between various demographic traits and psychological disorders. Female respondents had higher prevalence rates of anxiety than males (63.61 vs. 56.63%). The multivariate analysis did not show that females were more likely to develop anxiety. This finding is in contrast to a previous study performed in Saudi Arabia, which demonstrated that female HCWs were at greater risk of anxiety (46). However, we used a different screening tool and found that the younger age group had significantly higher prevalence rates of depression and anxiety than the older age group. A similar finding was observed in a Chinese study, which suggested that age is negatively associated with depression, anxiety, and insomnia, indicating that older age is a protective factor (47). In the bivariate analysis, we observed that married participants had lower rates of depression, anxiety, and stress. However, after adjusting for other factors, marital status was not associated with psychological disorders. A study of frontline HCWs in China found that the stress level was higher among married participants, likely due to the fear of transmitting the infection to one's spouse (48).

Our results indicated that geographic region was associated with depression and stress. Interestingly, the multivariate analysis shows that HCWs from the southern region were more likely to develop depression and stress. A previous study of HCWs in Saudi Arabia found that respondents from the central region had higher scores of depression and anxiety than those from other regions (34), but the study sample consisted of respondents from only three regions and did not include HCWs from the southern region. The findings of a Chinese study suggested that HCWs in Wuhan, the epicenter of the pandemic, were more likely to experience distress than those working outside Wuhan (49). Surely, it is expected that HCWs in locations with COVID-19 outbreaks would experience a greater psychological impact. However, the southern region of Saudi Arabia had fewer new and cumulative COVID-19 cases compared to the central or western regions. While it is unclear why HCWs from the southern region were more likely to suffer from depression and stress, this finding highlights the need to promote mental health and provide support services across the kingdom. Future research is warranted to address the variability in the psychological impact on HCWs from different geographic regions.

The findings revealed that HCWs with chronic illnesses had significantly higher prevalence rates of psychological disorders. In the multivariate analysis, we found that chronic disease was the strongest predictor of depression, anxiety, and stress. This finding corresponds with the literature, which suggests that people with chronic ailments are more prone to suffer from depression, anxiety, and stress (33, 35, 50, 51). This could be explained by the fact, established early in the pandemic, that people with chronic diseases are at greater risk of severe and potentially fatal COVID-19 disease (52). The results have also shown that knowing someone who died due to COVID-19 was a significant predictor of anxiety. Special attention should be paid to providing adequate personal protective supplies to HCWs with chronic illnesses.

Some limitations need to be considered when interpreting the findings. First, as this was a cross-sectional study, the causal relationship between various factors and psychological disorders could not be established, and we were unable to identify whether participants had pre-existing mental health issues that could have influenced the results. Second, we did not collect data on potential confounders, such as workload or social and organizational support, or whether participants were involved in the direct care of COVID-19 patients. Third, we used a non-probability sampling technique, which may have led to selection bias and limited generalizability of the findings. Nonetheless, our sample included HCWs from all regions of Saudi Arabia. Finally, the use of self-administered surveys could potentially increase response bias. Despite these limitations, our study provides valuable insight into decision-makers in healthcare institutions regarding how the COVID-19 crisis has affected HCWs.

## Conclusion

Physicians, pharmacists, and nurses alike have been deeply impacted by the COVID-19 crisis. The estimated prevalence rates of depression, anxiety, and stress among HCWs in Saudi Arabia were considerably high. This study identified certain populations who are at a greater risk of psychological disorders. Generally, factors associated with psychological disorders include having a chronic illness, being a nurse, knowing someone who died due to COVID-19, and being a healthcare worker from the southern region. Our study underscores the need to provide and promote support services for HCWs to mitigate the psychological impact of this pandemic.

## Data Availability Statement

The original contributions presented in the study are included in the article/supplementary material, further inquiries can be directed to the corresponding authors.

## Ethics Statement

The study was approved by the Scientific Research Ethics Committee at Taif University (42-0068) and the Institutional Review Board of MOH (472). The patients/participants provided their written informed consent to participate in this study.

## Author Contributions

AHA, MSA, FA, and AAA contributed to conception and design of the study. FA, AAlha, SFA, RA, and AAlz collected and organized the database. MSA, FA, and YA performed and validated the statistical analysis and wrote the statistical section. AHA, MSA, MAA, and AAA wrote the first draft of the manuscript. AB, HA, and YSA revised and edited the manuscript. All authors contributed to revising the manuscript and read and approved the submitted version.

## Funding

This research was funded by Taif University initiative for COVID-19 projects, Grant Number: 1-441-77.

## Conflict of Interest

The authors declare that the research was conducted in the absence of any commercial or financial relationships that could be construed as a potential conflict of interest.

## Publisher's Note

All claims expressed in this article are solely those of the authors and do not necessarily represent those of their affiliated organizations, or those of the publisher, the editors and the reviewers. Any product that may be evaluated in this article, or claim that may be made by its manufacturer, is not guaranteed or endorsed by the publisher.
